# Postacute Rehabilitation Impact on Functional Recovery Outcome and Quality of Life in Stroke Survivors: Six Month Follow-Up

**DOI:** 10.3390/medicina58091185

**Published:** 2022-08-30

**Authors:** Emir Bisevac, Milica Lazovic, Dejan Nikolic, Elvis Mahmutovic, Zana Dolicanin, Aleksandra Jurisic-Skevin

**Affiliations:** 1 Department of Physical Medicine and Rehabilitation, Faculty of Medical Sciences, University of Kragujevac, Svetozara Markovica 69, 34000 Kragujevac, Serbia; 2 Department of Biomedical Sciences, State University of Novi Pazar, 36300 Novi Pazar, Serbia; 3 Institute for Rehabilitation, 11000 Belgrade, Serbia; 4 Faculty of Medicine, University of Belgrade, 11000 Belgrade, Serbia; 5 Department of Physical Medicine and Rehabilitation, University Children’s Hospital, 11000 Belgrade, Serbia

**Keywords:** functional recovery, quality of life, stroke, ischemic, hemorrhagic, postacute rehabilitation

## Abstract

*Background and Objectives*: This study aimed to examine the impact of postacute rehabilitation duration on the outcome of the functional recovery and patients’ quality of life after the stroke. *Materials and Methods*: One hundred patients (52 females, 48 males, mean age: 66.5 ± 7.3; range 53 to 79 years) who experienced a stroke (50 with ischemic stroke (IS) and 50 with intracranial hemorrhage (ICH)) took part in the study. Patients (treated with postacute rehabilitation measures for six months) were examined after one, three, and six months of postacute rehabilitation. Functional independence was measured using the functional independence measure (FIM) test, while the EQ-5D-3L questionnaire was used to assess the quality of life. *Results*: Patients with ICH had a slightly lower FIM score (FIM motor = 29.8 ± 11.8; FIM cognitive = 14.4 ± 4.6) on admission compared to patients with IS (FIM motor = 41.8 ± 18.8; FIM cognitive = 18.7 ± 6.3), but, after six months of postacute rehabilitation, patients with ICH reached an approximate level of functional independence (FIM motor = 53.8 ± 14.4; FIM cognitive = 25.8 ± 4.7), as did patients with IS (FIM motor = 67.6 ± 16.4; FIM cognitive = 29.2 ± 4.0). The motor and cognitive FIM, as well as quality of life, was statistically significantly increased at all four measurement points (*p* < 0.001). Furthermore, there is a statistically significant connection between functional independence and quality of life at all tested times. *Conclusion*: Patients achieved the highest degree of functional independence after six months. Furthermore, our findings point out that inpatient rehabilitation as well as outpatient rehabilitation are effective in functionality and quality of life improvement after a stroke; thus, both should be emphasized and regularly implemented.

## 1. Introduction

Stroke is a clinically defined syndrome of acute, focal neurological deficit attributed to vascular injury of the central nervous system (CNS) [1]. It can leave permanent consequences, whereby patients become functionally dependent on others, with problems of a social nature arising as a result of physical or mental disability. Functional recovery is based on the restitution of brain tissue and on the relearning of and compensation for lost functions. Therefore, it is important that these patients be included in adequate postacute rehabilitation processes to increase the degree of functional independence [2].

There are two main types of stroke: ischemic stroke (IS) and hemorrhagic stroke (ICH) [3]. IS is the most common type of stroke. It occurs as a result of a blood vessel occlusion by an embolus or a thrombus [4]. ICH refers to bleeding in the brain, which occurs due to the rupture of a blood vessel. In order to prevent complications and permanent defects, early diagnosis is the key in stroke patients; however, distinguishing the type of stroke plays a crucial role in patient care [5]. It is advised that post-stroke treatment should start earlier, thus requiring that stroke rehabilitation trials be conducted within days post-stroke [6].

In the systematic analysis of GBD 2016 Stroke Collaborators, it was stated that the global burden of stroke is likely to remain high despite the sharp decrease in age-standardized mortality rates from 1990–2016 and with more than 80 million stroke survivors in 2016 [7]. More recent systemic analysis of GBD 2019 Stroke Collaborators pointed out that stroke remained the third leading Leve 3 cause of death and disability combined worldwide in 2019 [8]. In the study of Jankovic et al. conducted between October 2002 and September 2003 in Serbia for the reference year 2000, it was stated that, in males, stroke was the second cause of disability-adjusted life years (DALY) (17.9 per 1000) and for females the first cause of DALY (18.1 per 1000) [9].

The disability rates in post-stroke patients vary across different studies. In the study of Lv et al., [10] the three-years-post-stroke disability was 46.7%, while in the study of Cabral et al., [11] the disability rate was 18% in a third-year post-stroke. Furthermore, in the study of the et al., it was pointed out that stroke is the leading cause of the disability and disease burden globally [12]. Moreover, it is associated with greater care needs [12].

Well-organized rehabilitation, the process which enables individuals to regain physical, intellectual, psychological, sensory, and social functionality is key to acute, short-, and long-term recovery outcomes [13]. In the Guidelines for adult stroke rehabilitation and recovery, it was pointed out that comprehensive rehabilitation with adequate resources, doses, and duration is an essential aspect of stroke care [14]. Furthermore, it should be stated that the six-month rehabilitation treatment is very important for improving patients’ functional abilities [15].

More innovative treatments, particularly in stroke rehabilitation, are being considered. A randomized controlled trial of mobile game-based virtual reality program was shown to effectively promote the recovery of the upper extremities in patients with stroke [16]. Moreover, virtual reality exercises with the Nintendo Wii system might be useful adjunctive therapy with traditional therapy of stroke patients particularly for static and dynamic balance improvement [17].

The stroke was shown to incur substantial economic loss to the country, and 34% of total healthcare expenditure worldwide is spent on stroke [18]. In the systematic review of Rajsic et al., it was stressed that 27 European Union (EU) countries are estimated to spend 27 billion dollars yearly on stroke treatment and care [19]. In the United States of America (USA), the direct and indirect costs of stroke in 2008 were estimated at 65.5 billion dollars [20]. Furthermore, rehabilitation services were recognized as the main cost driver in post-stroke care [19].

According to the Serbia Health System Review, it was stated that the total expenditure on health for rehabilitation services in 2017 was 5.40% [21].

Although previous findings indicate that rehabilitation has a positive effect on the outcome of functional recovery and quality of life, further studies are needed in order to evaluate the optimal type, as well as amount, of therapy needed in this group of patients [22,23].

This study aimed to examine the impact of postacute rehabilitation duration on the outcome of functional recovery and patients’ quality of life after the stroke.

## 2. Materials and Methods

### 2.1. Study Design

The research was conducted as a prospective study at the Special Hospital for Progressive Muscular and Neuromuscular Diseases in Novi Pazar after obtaining the consent of the Ethics Committee (No: 872/2021, 20. October 2021). Data were collected by monitoring and examining patients who experienced a stroke, based on a neurological examination, magnetic resonance imaging (MRI), or computed tomography (CT) findings. Patients were examined at admission, after one month, after three months, and after six months of secondary rehabilitation. There were two groups of subjects, one group consisting of patients with IS, and the other group consisting of patients with ICH. All patients had the nature and purpose of the study, as well as the possibility of asking questions regarding participation in the study and withdrawing at any time without any consequences, carefully explained to them. All patients gave written informed consent to participate in the research.

### 2.2. Study Population

A total of 100 patients participated in the study, of which 50 (50%) had IS and 50 (50%) had ICH. Further risk factors were evaluated: high blood pressure, high cholesterol, diabetes mellitus, alcohol, coffee, and cigarette (current and ex-smokers) consumption. Inclusion criteria were being over 18 years old, having first experienced IS or ICH no earlier than one month from the start of the treatment, the patient’s ability to participate in a postacute rehabilitation program, the patient’s ability to communicate, and giving written consent to participate in the research. Exclusion criteria were being below 18 years old, having experienced IS or ICH before, the patient’s inability to speak, the refusal to give written consent, patients with some other neurological diseases that affected functional ability before the occurrence of stroke, the presence of pain worsened by exercising, the appearance of complications that do not allow further rehabilitation, and the occurrence of a second stroke during the monitoring of the patient and patient’s death.

### 2.3. Postacute Rehabilitation Program

The postacute rehabilitation treatment of study participants was conducted five days a week, and it included: motor learning with kinesitherapy, functional electrical stimulation (FES), and, where needed, static or dynamic orthoses, cognitive and speech rehabilitation, psychological and social integration, and patient and family education. It started after stabilization of the patients’ vital parameters and with the permission of a board-certified specialist in neurology, due to changes related to the CNS. Each patient was individually and multidisciplinarily assessed, and, based on that, an individual rehabilitation program was determined, which was adjusted daily to the patient’s condition. Patients were treated in the hospital for 3–4 weeks, and, after discharge from the hospital, they took a break of 3–4 weeks. After that, they were included in the outpatient program, which was in accordance with their needs, as individually and multidisciplinary assessed. Time intervals of 3–4 weeks alternated over 6 months, while the patients were monitored and examined in the meantime.

The kinesitherapy program was adapted to each patient, according to their functional abilities and usually lasted between 30 and 40 min per day. Passive or actively assisted exercises were performed in patients whose muscle activity was insufficient to move independently (Manual Muscle Testing (MMT)—grade 3 and below), while active or resistance exercises were performed in patients with greater muscle strength (MMT—grade 4 and above). Analgesic medications were applied in the case of occasional pain in the treated segment in some patients. Aids such as walkers, crutches, or canes were used in patients with balance disorders. Practicing walking was performed in the loom in front of a mirror in patients with gait problems. Occupational therapy was carried out using various props and equipment, with the main goal being improving mobility and transfer skills, as well as training hand movements, so that patients could use their hands correctly when eating, dressing, and maintaining personal hygiene as much as possible. In order to properly position or perform movements, static or dynamic orthoses were used in patients with balance disorders and reduced strength. Of the electrotherapeutic procedures, galvanic current (15 sessions lasting 10 min, once a day), interference current (15 sessions lasting 20 min, once a day), and FES were applied to strengthen muscles. Psychologists encouraged patients to manage illness-related emotions and to be optimistic and motivated about the rehabilitation. Speech therapists were in charge of solving speech problems and developing speech skills. Patients and their family members were educated about the correct way of living, to prevent complications and comorbidities.

### 2.4. Examination Procedure

The Functional Independence Measure test (FIM) was used to examine the functional ability of the patients. This test has two subscales: motor, which includes the domain of independent care, i.e., sphincter control, transfers, and movement, and cognitive, which involves communication and social cognition. Scores can be between 1 and 7, with the lowest indicating total assistance and the highest indicating complete independence. The total score of the FIM motor subscale can be between 13 and 91, while the score of the cognitive subscale can be between 5 and 35. The total score of the FIM scale can be between 18 and 126 [24].

The European Quality of Life—EuroQol EQ-5D-3L questionnaire, a general standardized quality-of-life indicator, was used to assess the quality of life. In our study, we used the descriptive system of EQ-5D-3L that is composed of five domains: mobility, self-care, usual activities, pain/discomfort, and anxiety/depression, at three severity levels (no problem, moderate problem, and severe problem) [25]. A designed questionnaire was used to collect socio-demographic characteristics that may have been relevant for the research results.

### 2.5. Statistical Analysis

Statistical analyses were performed using IBM SPSS version 24.0 software (IBM Corp. Armonk, NY, USA). The normality of the obtained values distribution was tested by Kolmogorov’s test, where the values of statistical significance are at the level of 0.05 and 2-tailed. A comparison of certain categories of attributive features frequency was performed using the Chi-square test. To determine the differences in repeated measurements, we analyzed the difference of variance with the ANOVA test (non-parametric Friedman), and then we used the Wilcoxon test of equivalent pairs. The analysis of the association of variables was performed using correlation analysis (Pearson or Spearman correlation). The change of FIM and EQ-5D-3L values after one, three, and six months of postacute rehabilitation in relation to admission is showed as a delta value (Δ). The descriptive statistics are presented as mean ± standard deviation (SD) or number (N) and frequency (%).

## 3. Results

Patient’s sociodemographic and clinical characteristics are shown in Table 1. From the tested patients, females were slightly more frequent (52%), married individuals composed 58%, high school education 38%, retired 25%, those with an average monthly income 67%, high blood pressure 71%, high cholesterol 46%, and diabetes mellitus 39%. A total of 63% of tested patients never used alcohol, 55% currently consume coffee, 60% belong to the group that currently use or were previously consuming cigarettes, and 81% currently use medicaments.

Descriptive statistic data for the FIM scale and the EQ-5D-3L questionnaire are shown in Table 2. Having applied the FIM-scale normality test, it was determined that the conditions had not been met, so statistical data processing used non-parametric tests. For the EQ-5D-3L, the requirements had been fulfilled only for data obtained during the three-month follow-up, but non-parametric tests were used nonetheless. The values of the FIM scores differed significantly on all tested occasions (at admission (*p* < 0.001), (one month (*p* < 0.012), three months (*p* = 0.034), and six months (*p* = 0.012)). For EQ-5D-3L, the values significantly differed at admission (*p* < 0.001), one month (*p* = 0.001), and six months (*p* = 0.007).

Applying the Chi-square test, it was examined whether there is a relationship between the type of stroke and certain sociodemographic variables, as shown in Table 3. Frequencies of gender (*p* = 0.230), the use of cigarettes (*p* = 0.870), alcohol (*p* = 0.409), and coffee (*p* = 0.601) did not significantly differ between stroke types (IS vs. ICH).

Results of the Spearman correlation analysis of stroke type and FIM, and stroke type and EQ-5D-3L are shown in Table 4. There is a statistically significant correlation of functional independence in all four moments of assessment of the functional state concerning the type of stroke (at admission (*p* < 0.001), one month (*p* < 0.001), three months (*p* = 0.001), and six months (*p* = 0.002)). In addition, the results show that there is a statistically significant correlation between the quality of life and the type of stroke at three measurements (at admission (*p* < 0.001), one month (*p* = 0.018), three months (*p* = 0.028), and six months (*p* = 0.049)).

For patients with IS, the Friedman test demonstrated a statistically significant difference between FIM values (total, motor and cognitive) at admission and FIM values (total, motor and cognitive) after one, three, and six months post admission, respectively, (*p* < 0.001). The same applies to those with ICH. The Bonferroni correction was used with a new statistical significance level of 0.025.

In further analysis, the Wilcoxon Signed Ranks Test showed a statistically significant improvement for the motor FIM and for the cognitive FIM subscales between admission and after one (*p* < 0.001), three (*p* < 0.001), and six (*p* < 0.001) months post admission of postacute rehabilitation, respectively, for both IS and ICH (Table 5).

The Wilcoxon Signed Ranks Test also revealed a statistically significant improvement in relation to the EQ-5D-3L questionnaire scores between admission and after one (*p* < 0.001), three (*p* < 0.001), and six (*p* < 0.001) months post admission of postacute rehabilitation, respectively, for both IS and ICH (Table 5).

The results of the correlation analysis, presented in Table 6, showed a statistically significant relationship between the values of the FIM test and the EQ-5D-3L questionnaire at admission (*p* < 0.001), one months (*p* < 0.001), three months (*p* < 0.001), and six months (*p* < 0.001). The correlation is positive and strong, which means that greater functional independence leads to a better quality of life for patients.

## 4. Discussion

The results of our study demonstrate that there is a statistically significant correlation of functional independence in all four time points of assessment with regards to the stroke type. Furthermore, our findings point out the presence of a statistically significant increase for the motor FIM and for the cognitive FIM scores between admission and after one, three, and six months post admission of postacute rehabilitation, respectively, for both IS and ICH. Moreover, we have demonstrated a statistically significant increase for EQ-5D-3L questionnaire scores between admission and after one, three, and six months post admission of postacute rehabilitation, respectively, for both IS and ICH. Additionally, a statistically significant relationship between the values of the FIM test and the EQ-5D-3L questionnaire at admission, one, three, and six months, respectively, was noticed in tested stroke patients.

From the 100 patients that were admitted to rehabilitation treatment, 53 (53%) of them completed the six-month postacute rehabilitation program and none of the studied participants had negative trends for FIM scores from admission up to 6 months of follow-up on all four testing time points. Unlike research dealing with similar topics, in our sample, there were slightly more women than men [26,27]. It is known that women experience stroke more often than men, but our study pointed out that there is no statistically significant association between gender and the type of stroke [28]. The average age of the subjects in our sample was 66.5 ± 7.3 years, which confirms that stroke occurs more often in older adults, as proven by recent studies [29,30]. Compared to older patients, there are discoveries about significantly better functional recovery and quality of life in the population between the ages of 15 and 45, including long-term outcomes [31]. Hence, we can say that our results refer to patients above that age limit, since all respondents were over 45 years old.

With advancements in the knowledge, there is a better understanding of the mechanisms related to stroke-induced paralysis; thus, the ability to stimulate the neuroplasticity is improving [32]. In the study of Mang et al., it was stated that aerobic exercise affects the brain by alterations in molecular signaling pathways, for example, the exercise-induced upregulation of brain-derived neurotrophic factor (BDNF) in the CNS [33]. The importance of BDNF is due to the fact that it is involved in neuroplastic changes and motor learning. Therefore, rehabilitation treatments aimed at motor learning-related neuroplasticity promotion are promising in functional outcome improvements in the poststroke period [33]. In addition to this, in patients who suffered stroke, it was shown that physical training through neuroplasticity promotion seems to have effects on functional motor recovery improvement. Such effects might be accelerated if physical training is combined with additional pharmacological treatment [34]. Furthermore, in poststroke survivors, for the affected upper limb motor recovery improvement, cortical brain stimulation combined with intense motor learning might show promise [35].

Regarding the impact of postacute rehabilitation on the outcome of functional recovery and quality of life, our findings pointed to the increase in total FIM scores on all tested occasions during postacute rehabilitation (one, three, and six months) when compared to the score at admission. Changes in the total FIM score at each measurement compared to the total FIM score at admission were statistically significant. In the study of Kamo et al., the authors evaluated the impact of the intensity of rehabilitation on the patients’ functionality level by determining the FIM score and proved that a longer rehabilitation program during the day is associated with higher values of the total FIM score [36]. Therefore, the individual assessment of a patient’s condition before and during postacute rehabilitation treatment, along with the adequate planning of the optimal rehabilitation program, is vital in order to achieve maximal functional restoration. Furthermore, it has been proven that, for stroke survivors and their families, a good and comprehensive postacute rehabilitation program is the key to a successful functional recovery that will allow them to reach the highest level of independence. Moreover, the outcome of postacute rehabilitation depends, to a great extent, on the cooperation of a large number of health workers within a multidisciplinary team [2].

Considering that the EQ-5D-3L questionnaire can be used as a reliable tool for quality-of-life measurement, particularly in stroke survivors [37], our results point out that there is a statistically significant difference between the scores of the EQ-5D-3L questionnaire after one month, three months, and six months of postacute rehabilitation in relation to the EQ-5D-3L questionnaire score measured at admission. These findings might lead to the assumption that a postacute rehabilitation program in stroke patients could have a beneficial impact on the improvement of the patient’s quality of life within 6 months of treatment.

Some authors believe exercise is one of the most effective and feasible rehabilitation strategies that significantly improve motor and cognitive abilities after IS, thanks to brain neuroplasticity [38]. In our case, patients who experienced IS achieved a significant recovery of both motor and cognitive abilities compared to admission, even after a month of postacute rehabilitation. Thus, it might be stressed that this shift indicates the importance of implementing postacute rehabilitation measures and procedures. Considering that the score of the motor and cognitive subscales increased significantly during each subsequent examination, and that the patients achieved the highest degree of functional independence after six months, it can be pointed out that postacute rehabilitation lasting less than six months allows patients to recover both motor and cognitive abilities to a certain degree and not reach the optimal functional potential during recovery. Patients with ICH in our sample had a slightly lower FIM score on admission and a more severe degree of functional impairment compared to patients with IS. However, after six months of rehabilitation, those with ICH reached an approximate level of functionality as patients with IS. Our findings are similar to the study conducted by Kelly et al., where the authors assessed the effect of rehabilitation on the outcome of functional recovery after IS and ICH [39]. A group of scientists researched the impact of combined cognitive and motor treatment and the effect of separately performing only motor or only cognitive treatment on the recovery of cognitive functions in patients after stroke. Analyzing the collected data, they proved that patients who had combined treatment achieved better results. In our case, a multidisciplinary team provided the patients with combined treatment, so we can agree with their claims, since the patients also had a significant recovery of cognitive abilities compared to admission [40].

It has been proven that, in addition to people who experience IS or ICH, family members and caregivers also suffer a certain level of burden, during which their quality of life decreases [41]. That is why it is very important to take measures to improve the quality of life of patients, which not only directly affects the reduction of the burden on families and caregivers, but also improve the quality of their lives. One study indicates the importance of postacute rehabilitation measures in order to achieve and maintain positive effects on quality of life [42]. Our results stress that quality of life increased gradually at each measurement compared to admission. Some authors believe that the quality of life in the first three months after a stroke depends on the patient’s upper limb strength [43]. Another study showed an association between quality of life and FIM scores at admission and after three months of stroke rehabilitation [44]. Analyzing our results, we can agree with that, because we proved, with a correlation analysis, that there is a statistically significant connection between functional independence and the quality of life of patients after a stroke. Capo-Lugo et al. proved that it is salient for patients who have experienced ICH to start implementing rehabilitation measures as early as possible to improve functional recovery and quality of life [45].

There are several limitations to this study. The first limitation is that the included patients were treated in only one rehabilitation center, so future research may include patients from more rehabilitation centers. An additional limitation refers to the number of participants, thus further studies on larger samples are needed. One more limitation of our study was not having data on the stroke localization, as well as using recanalization treatments or other drugs that can alter neuroplasticity or surgical procedures, particularly for ICH. So future research that will be dealing with a similar topic should collect that data as well.

## 5. Conclusions

Analyzing the collected data and comparing the obtained results, it can be concluded that postacute rehabilitation measures substantially impact patients’ conditions after a stroke. By following up for six months and examining on several occasions (after one, three and six months), it can be pointed out that including patients into an adequate postacute rehabilitation program increases their degree of functional independence. Furthermore, since there is a significant connection between functional independence and quality of life, it can be stressed that the longer duration of postacute rehabilitation, the greater functionality is achieved and thus a better quality of life for patients after a stroke.

## Figures and Tables

**Table 1:** Patient’s sociodemographic and clinical characteristics.

Tested Characteristics			Sample Size (*n* = 100)
Age (Year), Mean ± SD			66.5 ± 7.3
Gender, *n* (%)	Male		48 (48)
	Female		52 (52)
Marital status, *n* (%)	Not married		11 (11)
	Married		58 (58)
	Divorced		11 (11)
	Widow/widower		20 (20)
Level of education, *n* (%)	Primary school		36 (36)
	High school		38 (38)
	Higher education		15 (15)
	College		11 (11)
Employment status, *n* (%)	Unemployed		20 (20)
	Employed		19 (19)
	Housewife		18 (18)
	Farmer		18 (18)
	Retired		25 (25)
Monthly income, *n* (%)	Below average		23 (23)
	Average		67 (67)
	Above average		10 (10)
High blood pressure, *n* (%)	Yes		71 (71)
	No		29 (29)
High cholesterol, *n* (%)	Yes		46 (46)
	No		54 (54)
Diabetes mellitus, *n* (%)	Yes		39 (39)
	No		61 (61)
Alcohol, *n* (%)	Never used		63 (63)
	Currently using		3 (3)
	Previously used		34 (34)
Coffee, *n* (%)	Never used		7 (7)
	Currently using		55 (55)
	Previously used		38 (38)
Cigarettes, *n* (%)	Never used		40 (40)
	Currently using		24 (24)
	Previously used		36 (36)
Medicaments, *n* (%)	Never used		9 (9)
	Currently using		81 (81)
	Previously used		10 (10)
Children, *n* (%)	Yes		80 (80)
	No		20 (20)
Type of stroke, *n* (%)	Ischemic, N (%), *n* = 50	Male	27 (27)
		Female	23 (23)
	Hemorrhagic, N (%), *n* = 50	Male	21 (21)
		Female	29 (29)

SD—Standard deviation.

**Table 2:** Descriptive statistics for FIM and EQ-5D-3L questionnaire at different times of observation and results of test of normality.

Tested Measures	Mean ± SD	*p* *
FIM (admission)	47.1 ± 18.0	<0.001
FIM (one month)	59.5 ± 18.2	0.012
FIM (three months)	75.5 ± 20.1	0.034
FIM (six months)	88.2 ± 20.1	0.012
EQ-5D-3L (admission)	6.2 ± 1.9	<0.001
EQ-5D-3L (one month)	7.7 ± 2.2	0.001
EQ-5D-3L (three months)	9.2 ± 2.4	0.067
EQ-5D-3L (six months)	10.9 ± 2.4	0.007

* Kolmogorov–Smirnov test.

**Table 3:** Statistical interpretation of tested variables with regards to the stroke type.

Tested Variables		Stroke Type		*p* *
		IS	ICH	
		*n* (%)	*n* (%)	
Gender, *n* (%)	Male	27 (54)	21 (42)	0.230
	Female	23 (46)	29 (58)	
Cigarettes, *n* (%)	Never used	20 (40)	20 (40)	0.870
	Currently using	13 (26)	11 (22)	
	Previously used	17 (34)	19 (38)	
Alcohol, *n* (%)	Never used	29 (5)8	34 (68)	0.409
	Currently using	1 (2)	2 (4)	
	Previously used	20 (40)	14 (28)	
Coffee, *n* (%)	Never used	4 (8)	3 (6)	0.601
	Currently using	25 (50)	30 (60)	
	Previously used	21 (42)	17 (34)	

IS—ischemic stroke; ICH—intracranial hemorrhage; * Pearson Chi-Square test.

**Table 4:** Spearman correlation analysis between type of stroke and FIM, and type of stroke and EQ-5D-3L.

Tested Measures versus Stroke Type	Correlation Coefficient	*p*
FIM (admission)	−0.387	<0.001
FIM (one month)	−0.396	<0.001
FIM (three months)	−0.307	0.001
FIM (six months)	−0.438	0.002
EQ-5D-3L (admission)	−0.377	<0.001
EQ-5D-3L (one month)	−0.237	0.018
EQ-5D-3L (three months)	−0.220	0.028
EQ-5D-3L (six months)	−0.203	0.049

**Table 5:** Results of analyses for related samples and descriptive statistics of motor and cognitive FIM subscale and EQ-5D-3L in relation to the type of stroke.

Type of Stroke	Tested Measures and Time Points	Mean ± SD				*p* *
IS		FIM		FIM motor	FIM cognitive	
	At admission	54.5 ± 20.9	∆	41.8 ± 18.8	18.7 ± 6.3	-
	One month	68.3 ± 19.5	13.8	52.8 ± 18.3	22.7 ± 6.2	<0.001
	Three months	84.3 ± 21.0	29.8	62.4 ± 18.5	26.4 ± 5.8	<0.001
	Six months	96.6 ± 19.1	42.1	67.6 ± 16.4	29.2 ± 4.0	<0.001
ICH		FIM		FIM motor	FIM cognitive	
	At admission	39.6 ± 10.3	∆	29.8 ± 11.8	14.4 ± 4.6	-
	One month	50.6 ± 11.4	11	39.8 ± 12.9	18.3 ± 4.9	<0.001
	Three months	66.7 ± 14.8	27.1	51.1 ± 14.5	22.8 ± 5.0	<0.001
	Six months	79.5 ± 17.3	39.9	53.8 ± 14.4	25.8 ± 4.7	<0.001
IS		EQ-5D-3L				
	At admission	7.5 ± 2.7			∆	-
	One month	9.4 ± 3.0			1.9	<0.001
	Three months	11.0 ± 2.7			3.5	<0.001
	Six months	11.7 ± 1.9			4.2	<0.001
ICH		EQ-5D-3L				
	At admission	5.8 ± 1.7			∆	-
	One month	7.9 ± 2.3			2.1	<0.001
	Three months	9.7 ± 2.5			3.9	<0.001
	Six months	10.9 ± 2.6			5.1	<0.001

* Wilcoxon Signed Ranks test; IS—ischemic stroke; ICH—intracranial hemorrhage; SD—Standard deviation; ∆—value change.

**Table 6:** Correlation analysis between FIM and EQ-5D-3L.

		EQ-5D-3L (Admission)	EQ-5D-3L (One Month)	EQ-5D-3L (Three Months)	EQ-5D-3L (Six Months)
FIM (admission)	CC	0.664	-	-	-
	*p*	<0.001	-	-	-
FIM (one month)	CC	-	0.785	-	-
	*p*	-	<0.001	-	-
FIM (three months)	CC	-	-	0.776	-
	*p*	-	-	<0.001	-
FIM (six months)	CC	-	-	-	0.631
	*p*	-	-	-	<0.001

CC: Correlation coefficient.

## Data Availability

Study data is available upon request from first author.
